# Robust inference of positive selection on regulatory sequences in the human brain

**DOI:** 10.1126/sciadv.abc9863

**Published:** 2020-11-27

**Authors:** Jialin Liu, Marc Robinson-Rechavi

**Affiliations:** 1 Department of Ecology and Evolution, University of Lausanne, 1015 Lausanne, Switzerland.; 2 Swiss Institute of Bioinformatics, 1015 Lausanne, Switzerland.

## Abstract

A longstanding hypothesis is that divergence between humans and chimpanzees might have been driven more by regulatory level adaptations than by protein sequence adaptations. This has especially been suggested for regulatory adaptations in the evolution of the human brain. We present a new method to detect positive selection on transcription factor binding sites on the basis of measuring predicted affinity change with a machine learning model of binding. Unlike other methods, this approach requires neither defining a priori neutral sites nor detecting accelerated evolution, thus removing major sources of bias. We scanned the signals of positive selection for CTCF binding sites in 29 human and 11 mouse tissues or cell types. We found that human brain–related cell types have the highest proportion of positive selection. This result is consistent with the view that adaptive evolution to gene regulation has played an important role in evolution of the human brain.

## Teaser

A new method provides evidence that evolution of gene activity in the human brain was driven by natural selection.

## INTRODUCTION

It has long been suggested that changes in gene regulation have played an important role in human evolution and especially in the evolution of the human brain and behavior (*1*, *2*). Many human and chimpanzee divergent traits (*3*) cannot be explained by protein sequence adaptations. For example, there is little evidence to link protein sequence adaptations to traits related to cognitive abilities (*4*). Conversely, there is some evidence of brain-specific gene expression divergence in humans (*5*), which is consistent with a role of regulatory evolution. However, a central question remains open: Which regulatory changes were adaptive, if any? A major limitation in answering this is the lack of a robust model of neutral versus adaptive evolution for regulatory elements.

One approach to detect adaptive evolution on regulatory elements is to detect noncoding regions with lineage-specific accelerated evolutionary rates (*6*–*8*). For example, Gittelman *et al.* (*8*) found human accelerated regions close to genes annotated to terms such as brain or neuron development. A major caveat is that this acceleration may result from neutral mechanisms such as biased gene conversion (*9*) rather than from selection. A second approach is to use an MK (McDonald–Kreitman) test framework (*10*–*14*). This approach has two limitations. First, an expected neutral divergence to polymorphism ratio needs to be defined, whereas defining neutral sites for regulatory elements is difficult and can bias results (*15*). Second, it lacks power on individual elements, since many regulatory elements are short and present very few variable sites (*14*).

We have developed a new method to detect adaptive evolution of transcription factor binding sites (TFBSs) on the basis of predicted binding affinity changes. As a proof of principle, we first applied this method to well-known transcription factors, such as CEBPA and CTCF, in species triples focused on human, mouse, or fly. We validated it with three independent lines of evidence: Our evidence of positive selection is associated to higher empirical binding affinity, higher substitution-to-polymorphism ratio in sequence, and lower variance in expression of neighboring genes. Then, we used this method to detect positive selection of CTCF binding sites in 29 human tissues or cell types. We found the highest positive selection in brain samples, followed by male reproductive system. The same analysis in mouse found the highest positive selection in the lung, with no special signal in the brain. Thus, we provide evidence for adaptive evolution of gene regulation in the human brain.

## RESULTS

### Detecting positive selection on TFBSs

**Fig. 1:**
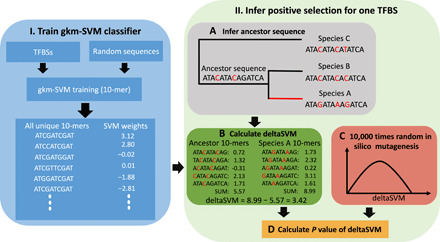
Illustration of the procedure for inferring positive selection. The method includes two parts. Part I (**left**) is the gapped *k*-mer support vector machine (gkm-SVM) model training. The gkm-SVM classifier was trained by using TFBSs as a positive training set and randomly sampled sequences from the genome as a negative training set. Then, SVM weights of all possible 10-mers, the contributions of prediction transcription factor binding affinity, were generated from the gkm-SVM. Part II (**right**) is the positive selection inference. The ancestor sequence was inferred from sequence alignment with a sister species (species B) and an outgroup (species C). Then, the binding affinity change (deltaSVM) of the two substitutions accumulated in the red branch leading to species A was calculated on the basis of the weight list. The significance of the observed deltaSVM was evaluated by comparing it with a null distribution of deltaSVM, constructed by scoring the same number of random substitutions 10,000 times.

We propose a computational model to detect positive selection on TFBSs, or any other elements for which we have experimental evidence similar to chromatin immunoprecipitation sequencing (ChIP-seq) (Fig. 1 and Materials and Methods). Briefly, a gapped *k*-mer support vector machine (gkm-SVM) classifier is trained on ChIP-seq peaks (here, TFBSs). This allows computing SVM weights of all possible 10-mers, which are predictions of their contribution to transcription factor binding affinity (*16*). We can then predict the binding affinity impact of substitutions by calculating deltaSVM, the difference of sum weights between two homologous sequences. We compare each empirical TFBS to an ancestral sequence inferred from alignment with a sister species and an outgroup.

Adaptive evolution on TFBSs is expected to push them from a suboptimal toward an optimal binding strength or from an old optimum to a new one (e.g., in response to changing environment). Thus, TFBSs evolving adaptively are expected to accumulate substitutions that consistently change the phenotype to stronger or to weaker binding, whereas TFBSs evolving under purifying selection are expected to accumulate substitutions that increase or diminish binding in approximately equal measure, around a constant optimum. This reasoning follows the principle of a sign test of phenotypes (*17*, *18*), although it uses the actual values and not just the sign. In practice, this should lead to a large absolute value of deltaSVM under adaptive selection. We estimate by randomization a *P* value specific to each individual TFBS and to its number of substitutions (see Materials and Methods). Thus, our method can infer the action of natural selection pushing a TFBS to a new fitness peak of either higher (positive deltaSVM) or lower (negative deltaSVM) binding affinity than its ancestral state.

### Detecting positive selection on liver TFBSs in *Mus musculus*

**Fig. 2:**
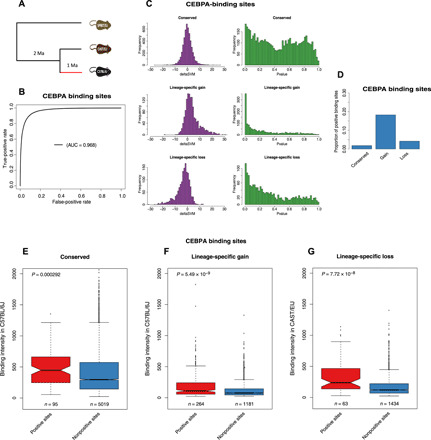
Mouse CEBPA binding sites study. (**A**) Topological illustration of the phylogenetic relationships between the three mouse species used to detect positive selection on CEBPA binding sites. We want to detect positive selection that occurred on the lineage of C57BL/6J after divergence from CAST/EiJ, as indicated by the red branch. Ma, million years. (**B**) Receiver operating characteristic (ROC) curve for gkm-SVM classification performance on CEBPA binding sites (fivefold cross-validation). The AUC value represents the area under the ROC curve and provides an overall measure of predictive power. (**C**) The left-hand graphs are the distributions of deltaSVM. The right-hand graphs are the distributions of deltaSVM *P* values (test for positive selection). (**D**) Proportion of CEBPA binding sites with evidence of positive selection. (**E** to **G**) The number of binding sites in each category is indicated below each box. The *P* values from a Wilcoxon test comparing categories are reported above boxes. Positive sites are binding sites with evidence of positive selection (deltaSVM *P* value <0.01). (E) Conserved binding sites. (F) Lineage-specific gain binding sites. (G) Lineage-specific loss binding sites. We compare the binding intensity from CAST/EiJ, as an approximation for ancestral binding intensity, between positive loss binding sites and nonpositive loss binding sites.

We first applied our method to a large set of TFBSs in the liver of three mouse species (*M. musculus domesticus* C57BL/6J, *M. musculus castaneus* CAST/EiJ, and *M. spretus* SPRET/EiJ), identified by ChIP-seq for three liver-specific transcription factors, CEBPA, FOXA1, and HNF4A (*19*). We inferred positive selection on the lineage leading to C57BL/6J after divergence from CAST/EiJ (Fig. 2A). For the sake of simplicity, we only present the results of CEBPA in the main text; results are consistent for FOXA1 and HNF4A (Supplementary Materials). We first trained a gkm-SVM on 41,945 CEBPA binding sites in C57BL/6J (see Materials and Methods). The gkm-SVM very accurately separates CEBPA binding sites and random sequences (Fig. 2B). On the basis of the experimental ChIP-seq peaks in the three species, using SPRET/EiJ as an outgroup, we identified three categories of CEBPA binding sites: conserved in all three species (“conserved,” 24,280 sites), lineage-specific gain in C57BL/6J (“gain,” 6304 sites), and lineage-specific loss in C57BL/6J (“loss,” 6692 sites). On the basis of whole-genome pairwise alignments of C57BL/6J to CAST/EiJ and to SPRET/EiJ, we derived the substitutions accumulated on the C57BL/6J lineage for each CEBPA binding site (see Materials and Methods). We only kept binding sites with at least two substitutions, leading to 5114, 1445, and 1497 TFBSs for conserved, gain, and loss categories, respectively. For each binding site, we calculated a deltaSVM value and inferred its significance by random in silico mutagenesis (see Materials and Methods).

We plot the distributions of deltaSVMs and their corresponding *P* values for each binding site evolutionary category (Fig. 2C). As expected, the distribution of deltaSVMs is symmetric for conserved, has a skew toward positive deltaSVMs for gain, and a skew toward negative deltaSVMs for loss. These results confirm that the gkm-SVM–based approach can accurately predict the effect of substitutions on transcription factor binding affinity change. For the distribution of *P* values, in all binding site categories, there is a skew of *P* values near zero, indicating some signal of positive selection. Gain has the most skewed distribution of *P* values toward zero. Hereafter, we will use 0.01 as a significant threshold to define positive selection, but results are robust to different thresholds (see the “Validation based on ChIP-seq binding intensity” section). This identifies almost 20% of gain having evolved under positive selection (Fig. 2D), relative to 4% of loss and 2% of conserved. Random substitutions tend to decrease the binding affinity rather than increase it (fig. S1), because it is easier to break a function than to improve it. Thus, our method could be biased toward reporting as positive sites with more left-shifted null distributions. However, this is not the case (fig. S2).

In summary, we found widespread positive selection driving the gain of CEBPA binding sites. We also found some evidence of positive selection driving loss or increase in binding affinity in some conserved sites. For the other two transcription factors (FOXA1 and HNF4A), we found very consistent patterns (figs. S3 and S4).

### Validation based on ChIP-seq binding intensity

We expect that conserved or gained sites, which evolved under positive selection with positive deltaSVM, should have increased binding affinity. Thus, the positive binding sites (PBSs) should have higher binding affinity than nonpositive selection binding sites (non-PBSs) in the focal species C57BL/6J. This is indeed the case (Fig. 2, E and F). In addition, conserved TFBSs have higher activity than recently evolved ones (“gain”). For loss, however, the PBSs have a strong decrease in binding affinity, so we expect higher binding affinity of PBSs in the ancestor. Using CAST/EiJ as an approximation for ancestor binding affinity, this is indeed the case (Fig. 2G). Results are also consistent with different *P* value thresholds (fig. S5). We performed the same validations in FOXA1 and HNF4A, with consistent results (fig. S6).

### Validating the inference of positive selection with human liver TFBSs

**Fig. 3:**
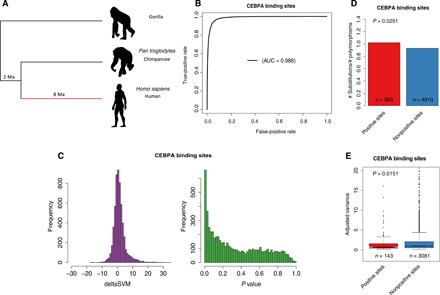
Human CEBPA binding site study. (**A**) Topological illustration of the phylogenetic relationships between human, chimpanzee, and gorilla. We detected positive selection that occurred on the lineage of human after divergence from chimpanzee, as indicated by the red branch. (**B**) ROC curve for gkm-SVM classification performance on CEBPA binding sites (fivefold cross-validation). The AUC value represents the area under the ROC curve and provides an overall measure of predictive power. (**C**) The left graph is the distribution of deltaSVM. The right graph is the distribution of deltaSVM *P* values (test for positive selection). (**D**) Ratio between the number of substitutions and the number of polymorphisms [single-nucleotide polymorphisms (SNPs)] for CEBPA binding sites. Positive sites are binding sites with evidence of positive selection (deltaSVM *P* value <0.01). The *P* value from Fisher’s exact test is reported above the bars. (**E**) Comparison of expression variance (adjusted variance) of putative target genes (closest gene to a TFBS) between positive sites and nonpositive sites. The number of binding sites in each category is indicated below each box. The *P* values from a Wilcoxon test comparing categories are reported above boxes. Positive sites are binding sites with evidence of positive selection (deltaSVM *P* value <0.01).

To further validate our method, we took advantage of the abundant population genomics transcriptomics data in humans. We inferred positive selection of CEBPA binding sites in the human lineage after divergence from chimpanzee, with gorilla as outgroup (Fig. 3A). As in mouse, the gkm-SVM trained from 15,806 CEBPA binding sites in human can very accurately separate TFBSs and random sequences (Fig. 3B). The distribution of deltaSVMs is slightly asymmetric, with a higher proportion of positive values (Fig. 3C). This is because these binding sites contain both conserved and gain, but no loss (since we detect only in the focal species). On the basis of the distribution of *P* values, 7.5% of CEBPA binding sites are predicted to have evolved adaptively in the human lineage.

Using the MK framework (*10*), we predict that PBSs should have higher substitution-to-polymorphism ratios than non-PBSs. Note that we do not need to define neutral sites a priori. As expected, we found that the PBSs have a significantly higher ratio of fixed nucleotide changes between human and chimpanzee to polymorphic sites in human than non-PBSs (Fig. 3D). This is an external validation that our method detects positive selection, as the input did not contain any information about polymorphism.

Besides a higher substitution-to-polymorphism ratio, we also expect that the expression of PBS putative target genes (see Materials and Methods) should be more conserved among human populations. If the expression of PBS target genes is an adaptive trait in humans, further changes in expression will reduce fitness. Moreover, recent adaptive sweeps are expected to have reduced variability for the regulation of these genes. As expected, we found that PBS target genes have significantly lower expression variance (adjusted variance, controlling for the dependency between mean and variance; see Materials and Methods) across human populations than non-PBS target genes (Fig. 3E).

Thus, results from different sources of information support the expectations of our PBS predictions. We performed the same analyses in HNF4A, and results are consistent (fig. S7). These results strongly suggest that our method is detecting real adaptive evolution signals.

### Detecting positive selection of TFBSs in *Drosophila melanogaster*

By using an MK test framework (*10*), Ni *et al.* (*20*) detected signatures of adaptive evolution on CTCF binding sites in *D. melanogaster*. They reported that positive selection has shaped CTCF binding evolution and that newly gained binding sites show a stronger signal of positive selection than conserved sites. We applied our method to the same data as used in Ni *et al.* (*20*). We detected positive selection in the *D. melanogaster* lineage after divergence from *Drosophila simulans* (fig. S8, A and B). Consistent with the findings of Ni *et al.* (*20*), we observed widespread positive selection for both conserved and gain (fig. S8C). In addition, the gain has a higher proportion of positive selection than conserved (fig. S8D). As Ni *et al.* (*20*) did not report specific sites, we cannot compare results more precisely. For lineage-specific loss binding sites, however, we did not detect any signal of positive selection (fig. S8C). The proportion of positive selection in *D. melanogaster* is much higher than in *M. musculus.* For example, we find almost 40% of gain under positive selection in *D. melanogaster*, twice the proportion in *M. musculus*. It should be noted that different transcription factors and tissues were used, which complicates direct comparison.

### Adaptive evolution of CTCF binding sites across tissues in human

To test whether there is stronger adaptive evolution of gene regulation in some human tissues, we applied our method to 80,074 CTCF binding sites across 29 adult tissues or primary cell types (hereafter “cell types”; see table S2). We chose CTCF because it was the factor with the largest number of tissues or primary cell types studied in a consistent manner by the ENCODE (Encyclopedia of DNA Elements) consortium (*21*, *22*). CTCF is well known as a transcriptional repressor, but it is also involved in transcriptional insulation and chromatin architecture remodeling (*23*). The gkm-SVM model trained from one cell type can accurately predict the binding sites in another cell type, and the model trained with all CTCF binding sites has better performance than the model trained with cell type–specific binding sites (fig. S9). Thus, we used a general gkm-SVM rather than different models for different cell types.

**Fig. 4:**
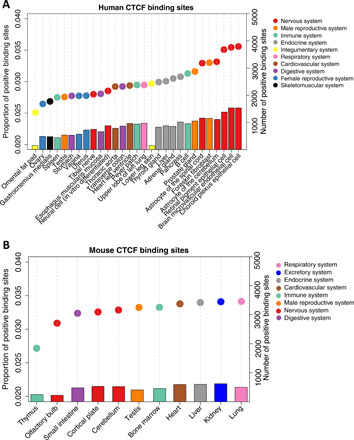
Proportion of positive CTCF binding sites in different tissues or cell types. PBSs are binding sites with evidence of positive selection (deltaSVM *P* value <0.01). Colors correspond to broad anatomical systems. (**A**) CTCF binding sites in 29 human tissues or cell types. (**B**) CTCF binding sites in 11 mouse tissues.

We detected 3.52% of PBSs for adaptation on the human lineage (fig. S10A). We found that PBSs have higher substitution-to-polymorphism ratio than non-PBSs (fig. S11). In addition, PBSs are associated with a lower number of active cell types (fig. S12A) than non-PBSs, consistent with the prediction that pleiotropy can limit adaptive evolution (*24*). We ranked cell types according to the proportion of binding sites that exhibit statistically significant evidence of positive selection. Brain-related cell types have a higher proportion of positive selection than other cell types (Fig. 4A). This pattern is consistent if we only use tissue-specific CTCF binding sites (fig. S13A). Choroid plexus epithelial cell, brain microvascular endothelial cell, and retinal pigment epithelial cell have notably high PBS frequencies. Non–brain-related nervous system cell types do not share this high positive selection, nor does in vitro differentiated neural cell, which may reflect that they do not preserve the signal of specific in vivo differentiated cells. Notably, these brain-related cell types also have a higher fraction of substitutions fixed by positive selection (see Materials and Methods) than other cell types, except lower leg skin (fig. S14).

To check whether our test could be too liberal or conservative for some sites, we first analyzed the substitution rate of all possible substitutions and their corresponding affinity change (deltaSVM) in human CTCF binding sites. We split all substitutions into two categories: substitutions on CpG and substitutions not on CpG. Within each category, we found, as expected, that the transition rate is much higher than the transversion rate, but we did not find a trend for specific substitution types to strengthen or weaken binding affinity (figs. S15A and S16). Between categories, we found that there is generally higher substitution rate on CpG, again as expected. Substitutions on CpG tend to weaken binding affinity (figs. S15A and S16), indicating that our test could be conservative for sites with CpG substitutions. Second, we checked whether neighboring substitutions (dinucleotide substitutions) have a general tendency to change affinity in the same direction. Indeed, this is the case (fig. S15B), suggesting that our test could be too liberal or too conservative for dinucleotide substitutions, depending on the direction of affinity change.

To check whether these biases (substitutions on CpG and dinucleotide substitutions) affect the pattern we found, first, we split all CTCF binding sites into two categories: sites with neither CpG substitutions nor dinucleotide substitutions and sites with either CpG substitutions or dinucleotide substitutions. For both categories, the proportion of positive selection binding sites (PBSs) detected is highly correlated with the original pattern (fig. S17, A and B). In addition, as expected, there is a higher proportion of PBSs for sites without substitutions on CpG, cofirming that our test is conservative for sites with CpG substitutions. Second, we both excluded all CpG sequences and dinucleotide substitution sequences from all binding sites, and we integrated the transition and transversion rate (4:1, estimated from fig. S15A) into our null model. Patterns of results were very robust to these changes (fig. S17C).

To test whether the high regulatory adaptive evolution in brain is general to mammals, we performed the same analysis on CTCF binding data from 11 mouse adult tissues (table S2 and fig. S18). We investigated adaptive evolution in the *M. musculus* branch after divergence from *M. spretus*, a similar evolutionary divergence to that between human and chimpanzee (*5*). Similarly to human, we detected 3.54% binding sites that evolved under positive selection (fig. S10B) and found PBSs associated with a lower number of active cell types (fig. S12B). However, no tissue type had especially high adaptive evolution, and brain-related tissues were among the lowest (Fig. 4B). When restricting to tissue-specific CTCF binding sites, lung has notably high adaptive evolution (fig. S13B).

## DISCUSSION

### A robust test for positive selection on regulatory elements

Detecting positive selection on regulatory sequences has long been a difficult problem (*15*). Nearby noncoding regions are often used as a neutral reference (*13*, *14*, *25*), but this neutrality is difficult to establish. Our approach does not require defining a priori neutral sites but instead considers the effects of variation on activity (*26*–*28*). Moreover, positive selection on a background of negative selection might not elevate the evolutionary rate above the neutral expectation, yet consistent changes in binding affinity can still be detectable. Indeed, the TFBSs of cell types detected under selection do not necessarily evolve faster (fig. S19). In principle, our method can also be applied to other genomic regions for which experimental peaks are available, such as open chromatin regions or histone modification regions.

Because positive selection on regulatory sequences is difficult to determine, it is important to validate our predictions with independent evidence. The most important validation is that predictions made independently of population data verify the expectations of higher substitution-to-polymorphism ratio (Fig. 3D). Both this and the lower expression variance of neighboring genes (Fig. 3E) are consistent with the prediction that positive selection will increase divergence but remove polymorphism (*10*) and that recently selected phenotypes will be under stronger purifying selection. Moreover, binding affinity change occurs in the direction predicted by our model (Fig. 2, E to G), and we can verify the prediction that pleiotropy limits adaptation (fig. S12) (*24*).

Despite its advantages, our method can still be improved. For example, in the null model of sequence evolution, we assume independent mutation patterns at each base pair (bp) site and a uniform mutation rate over all sites. However, both mutation rate and pattern can depend on neighboring nucleotides (*29*). These limitations of our null model might explain why the observed *P* values do not quite follow the expected uniform distribution for high values.

### Importance of regulatory adaptation on human brain evolution

Our results support the long proposed importance of adaptive regulatory changes in human brain evolution (*1*). They are consistent with accelerated gene expression evolution in the human brain, but neither in human blood or liver nor in rodents, from Enard *et al.* (*5*). Previous studies on human regulatory sequence evolution reported acceleration in brain-related functions but could not demonstrate adaptive evolution nor direct activity in the brain (*5*–*8*, *25*). The reported link between human accelerated regions and function was very indirect, depending both on the attribution of a region to the closest gene and on the functional annotation of that gene.

The brain-related cell types for which we detect a high proportion of positive selection are functionally related with cognitive abilities. For example, for astrocyte, abnormal astrocytic signaling can cause synaptic and network imbalances, leading to cognitive impairment (*30*). In addition, for choroid plexus epithelial cell, its atrophy has been reported to be related with Alzheimer’s disease (*31*).

While we did not find a similar pattern by applying the same analysis to mouse, it is not possible yet to conclude to a human- or primate-specific pattern. Indeed, the mouse analyses have two potential caveats. First, for the olfactory bulb and cortical plate in the mouse analyses, there are no corresponding anatomical structures in the human analyses. It is an open question whether the human olfactory bulb and cortical plate also have high adaptation. Second, the human analyses were based on ChIP-seq at cell type level, but the mouse analyses were based on ChIP-seq at tissue level. In mouse, the astrocyte in cerebellum may also have high adaptation like the astrocyte in human, but the signal might be diluted by other cell types in cerebellum.

### Regulatory adaptation differs between tissues

Outside of brain cell types, we found that male reproduction system (prostate and foreskin) has higher adaptive regulatory evolution than female reproduction system (ovary, uterus, and vagina). This is consistent with the observation of high adaptive sequence evolution in human male reproduction (*32*, *33*) and probably caused by sexual selection–related selective pressures, such as sperm competition. However, testis has a relatively low proportion of adaptive evolution, similar to ovary. This suggests that the high expression divergence in testis (*34*) is mainly caused by relaxed purifying selection, maybe due to the role of transcription in testis for “transcriptional scanning” (*35*). Outside of the brain, the top adaptive regulatory evolution systems seem to be the same as found for adaptive protein evolution, i.e., male reproduction, immune, and endocrine systems (*32*, *36*–*38*). The high fraction of substitutions fixed by positive selection in the skin is interesting (fig. S14), since the skin is both involved in defense against pathogens and has evolved specifically in the human branch with loss of fur (*39*). The lack of adaptive protein sequence evolution despite high adaptive regulatory evolution might be related to selective pressure on proteins in the brain (*40*, *41*).

## MATERIALS AND METHODS

### Mutagenesis for positive selection

#### Training of the gkm-SVM

gkm-SVM is a method for regulatory DNA sequence prediction by using *k*-mer frequencies (*42*). For the gkm-SVM training, we followed the approach of Lee *et al.* (*16*). First, we defined a positive training set and its corresponding negative training set. The positive training set is ChIP-seq narrow peaks of transcription factors. The negative training set is an equal number of sequences, which randomly sampled from the genome with matched the length, GC (guanine-cytosine) content, and repeat fraction of the positive training set. This negative training set was generated by using “genNullSeqs,” a function of gkm-SVM R package (*43*). Then, we trained a gkm-SVM with default parameters except −l = 10 (meaning, we use 10-mer as feature to distinguish positive and negative training sets). The classification performance of the trained gkm-SVM was measured by using receiver operating characteristic (ROC) curves with fivefold cross-validation. The gkm-SVM training and cross-validation were achieved by using the “gkmtrain” function of “LS-GKM: A new gkm-SVM software for large-scale datasets” (*44*). For details, please check https://github.com/Dongwon-Lee/lsgkm.

#### Generate SVM weights of all possible 10-mers

The SVM weights of all possible 10-mers were generated by using the “gkmpredict” function of “LS-GKM.” The positive value means increasing binding affinity, the negative value means decreasing binding affinity, and the value close to 0 means functionally neutral.

#### Infer ancestor sequence

The ancestor sequence was inferred from sequence alignment with a sister species and an outgroup.

#### Calculate deltaSVM

We calculated the sum of weights of all 10-mers for ancestor sequence and focal sequence, respectively. The deltaSVM is the sum weights of focal sequence minus the sum weights of ancestor sequence. The positive deltaSVM indicating substitutions increased the binding affinity in the focal sequence, vice versa.

#### Generate empirical null distribution of deltaSVM

First, we counted the number of substitutions between the ancestor sequence and the focal sequence. Then, we generated a random pseudo-focal sequence by randomly introducing the same number of substitutions to the ancestor sequence. Last, we calculated the deltaSVM between the pseudo-focal sequence and the ancestor sequence. We repeated the above processes 10,000 times to get 10,000 expected deltaSVMs.

#### Calculate P value of deltaSVM

For lineage-specific gain TFBSs, the *P* value was calculated as the probability that the expected deltaSVM is higher than the observed deltaSVM (higher-tail test). For lineage-specific loss TFBSs, the *P* value was calculated as the probability that the expected deltaSVM is lower than the observed deltaSVM (lower-tail test). For conserved TFBSs, we primarily focused on selection to increase binding affinity, and thus, we performed higher-tail test. The motivation for this is that when we have ChIP-seq data in only one species, which is the most common case, the observed peaks are a mix of conserved and gained sites, and thus, very little signal of decrease of binding is expected. The *P* value can be interpreted as the probability that the observed deltaSVM could arise by chance.

### Mouse validation analysis

#### ChIP-seq data

The narrow ChIP-seq peaks and their corresponding intensity (normalized read count) datasets of three liver-specific transcription factors (CEBPA, FOXA1, and HNF4A) in three mouse species (C57BL/6J, CAST/EiJ, and SPRET/EiJ) were downloaded from www.ebi.ac.uk/research/flicek/publications/FOG09 [accessed in May 2018; (*19*)]. Peaks were called with SWEMBL (https://github.com/stevenwilder/SWEMBL). To account for both technical and biological variabilities of peak calling, Stefflova *et al.* (*19*) carried out the following approaches. For each transcription factor in each species, they first called three sets of peaks: one for each replicate (replicate peek) and one for a pooled dataset of both replicates (pooled peak). Then, the peaks detected from the pooled dataset were used as a reference to search for overlaps in the two other replicates. When a pooled peak overlapped with both replicate peeks (at least 1-bp overlap), it was kept for downstream analyses. For the number of peaks and average peak length, please check table S1.

#### Peak coordinates transfer

On the basis of pairwise genome alignments between C57BL/6J and CAST/EiJ or SPRET/EiJ, Stefflova *et al.* (*19*) transferred the coordinates of ChIP-seq peaks in both CAST/EiJ and SPRET/EiJ to its corresponding coordinates in C57BL/6J.

#### Sequence alignment files

The sequence alignment files between C57BL/6J and CAST/EiJ or SPRET/EiJ were downloaded from www.ebi.ac.uk/research/flicek/publications/FOG09 [accessed in May 2018; (*19*)].

#### Define different types of binding sites

##### *Conserved binding sites*.

The conserved binding sites were defined as peaks in C57BL/6J, which have overlapping peaks (at least 1-bp overlap) in the other two species by genome alignment.

##### *Lineage-specific gain binding sites*.

The lineage-specific gain binding sites defined as peaks in C57BL/6J with no overlapping peaks (at least 1-bp overlap) in the other two species.

##### *Lineage-specific loss binding sites*.

The lineage-specific loss binding sites defined as peaks in CAST/EiJ, which have overlapping peaks in SPRET/EiJ but not in C57BL/6J.

### Human validation analysis

#### ChIP-seq data

The narrow ChIP-seq peak datasets of two liver-specific transcription factors (CEBPA and HNF4A) in human were downloaded from www.ebi.ac.uk/research/flicek/publications/FOG01 [accessed in October 2018; (*45*)]. Peaks were called with SWEMBL (https://github.com/stevenwilder/SWEMBL). Negligible variation was observed between the individuals in terms of peak calling, so Schmidt *et al.* (*45*) pooled replicates into one dataset for peak calling.

#### Sequence alignment files

The pairwise whole-genome alignments between human and chimpanzee or gorilla were downloaded from http://hgdownload.soe.ucsc.edu/downloads.html (accessed in December 2018).

#### Single-nucleotide polymorphism data

Over 36 million single-nucleotide polymorphisms (SNPs) for 1092 individuals sampled from 14 populations worldwide were downloaded from phase 1 of the 1000 Genomes Project (ftp://ftp.1000genomes.ebi.ac.uk/vol1/ftp/phase1/analysis_results/integrated_call_sets/) [accessed in December 2018; (*46*)]. As suggested by Luisi *et al.* (*47*), we only used SNPs of a subset of 270 individuals from YRI (Yoruba in Ibadan), CEU (Utah residents with Northern and Western European ancestry), and (Han Chinese in Beijing) populations.

#### Liver expression data

The library site normalized expression data of 175 livers were downloaded from The Genotype-Tissue Expression (GTEx) project https://gtexportal.org/home/ [Release V7, accessed in December 2018; (*48*)]. We further transformed it with log_2_.

#### Putative target genes of TFBSs

We assigned the nearest gene to each TFBS as its putative target gene.

#### Adjusted variance

There is a very strong dependency between mean and variance for gene expression (fig. S20A). To remove this dependency, as previously proposed (*49*, *50*), we calculated the adjusted variance. Specifically, we fitted a polynomial model to predict the variance from the mean in the log space. We increased the degrees of the model until there was no more significant improvement [tested with analysis of variance (ANOVA), *P* < 0.05 as a significant improvement]. The adjusted variance is the ratio of the observed variance over the variance component predicted by the mean expression level. After this adjustment, there is no correlation between mean and variance (fig. S20B).

### Fly validation analysis

#### ChIP-seq data

The narrow ChIP-seq peaks of transcription factor CTCF in three drosophila species (*D. melanogaster*, *D. simulans*, and *Drosophila yakuba*) were downloaded from www.ncbi.nlm.nih.gov/geo/query/acc.cgi?acc=GSE24449 [accessed in January 2019; (*20*)]. Peaks were called with QuEST (*51*) at a false discovery rate <1%. We obtained 2182, 2197, and 2993 peaks with average length of 243, 240, and 201 bp for *D. melanogaster*, *D. simulans*, and *D. yakuba*, respectively.

#### Peak coordinates transfer

The peaks identified in *D. simulans* and *D. yakuba* were translated onto *D. melanogaster* coordinates by using pslMap (*52*).

#### Sequence alignment files

The pairwise whole-genome alignments between *D. melanogaster* and *D. simulans* or *D. yakuba* were downloaded from http://hgdownload.soe.ucsc.edu/downloads.html (accessed in January 2019).

#### Define different types of binding sites

These were defined as in mouse, using *D. melanogaster* versus *D. simulans* and *D. yakuba*.

### Human CTCF analysis

#### ChIP-seq data

The narrow ChIP-seq peaks of transcription factor CTCF across 29 tissues or cell types (table S2) were downloaded from ENCODE (*21*). We did not use ChIP-seq datasets from cell lines and only kept ChIP-seq datasets from tissues and primary cells. Briefly, peaks were called with MACS (model-based analysis of ChIP-Seq) (*53*) separately for each replicate. Irreproducible discovery rate (IDR) analysis was then performed (*54*). Final peaks are the set of peak calls that pass IDR at a threshold of 2%. Peaks identified in different tissues or cell types were integrated by intersecting all peaks across datasets, with at least 1-bp overlap used as the merge criteria. Overall, we obtained 118,970 merged peaks.

#### Sequence alignment files

The pairwise whole-genome alignments between human and chimpanzee or gorilla were downloaded from http://hgdownload.soe.ucsc.edu/downloads.html (accessed in December 2018).

#### Proportion of substitutions fixed by positive selection

We calculated the proportion of substitutions fixed by positive selection, a measure of effect size of selection, under the MK test framework (*10*, *55*)

$$\alpha =1-\frac{D\text{np}Pp}{DpP\text{np}}$$

*D*np is the substitution number in non-PBSs, *P*p is the polymorphism number in PBSs, *D*p is the substitution number in PBSs, and *P*np is the polymorphism number in non-PBSs.

#### Estimate substitution rate

The substitution rate, for example C→T, was estimated as the number of C→T divided by the number of nucleotide C in the ancestor sequence.

### Mouse CTCF analysis

#### ChIP-seq data

The narrow ChIP-seq peaks of transcription factor CTCF across 11 tissues (table S2) were downloaded from ENCODE (*21*). Briefly, peaks were called with MACS (*53*) separately for each replicate. IDR analysis was then performed. Final peaks are the set of peak calls that pass IDR at a threshold of 2%. Peaks identified in different tissues/cell types were integrated by intersecting all peaks across datasets, with at least 1-bp overlap used as the merge criteria. Overall, we obtained 112,657merged peaks.

#### Sequence alignment files

The sequence alignment file between C57BL/6J and SPRET/EiJ (please check the “Mouse validation analysis” section in Materials and Methods). The sequence alignment files between C57BL/6J and Caroli/EiJ were downloaded from www.ebi.ac.uk/research/flicek/publications/FOG09 [accessed in May 2018; (*19*)].

## Supplementary Material

http://advances.sciencemag.org/cgi/content/full/6/48/eabc9863/DC1

Adobe PDF - abc9863_SM.pdf

Robust inference of positive selection on regulatory sequences in the human brain

## SUPPLEMENTARY MATERIALS

Supplementary material for this article is available at http://advances.sciencemag.org/cgi/content/full/6/48/eabc9863/DC1
