# Supplementary material for: Robust inference of positive selection on regulatory sequences in the human brain
Source: Sci Adv. 2020 Nov 27;6(48):eabc9863. doi: 10.1126/sciadv.abc9863 (PMC7695467; doi:10.1126/sciadv.abc9863)
Supplement: http://advances.sciencemag.org/cgi/content/full/6/48/eabc9863/DC1 [file supp_6_48_eabc9863__index.html]

Science Advances | Science AdvancesAAASSearchScience AdvancesMenu

## Supplementary Materials

# Robust inference of positive selection on regulatory sequences in the human brain

Jialin Liu, Marc Robinson-Rechavi

Download Supplement

**This PDF file includes:**

- Figs. S1 to S20
- Tables S1 and S2

**Files in this Data Supplement:**

- Adobe PDF - abc9863\_SM.pdf
