# Supplementary material for: Robust inference of positive selection on regulatory sequences in the human brain
Source: Sci Adv. 2020 Nov 27;6(48):eabc9863. doi: 10.1126/sciadv.abc9863 (PMC7695467; doi:10.1126/sciadv.abc9863)
Supplement: http://advances.sciencemag.org/cgi/content/full/6/48/eabc9863/DC1 [file supp_6_48_eabc9863__1.pdf]

Science Advances
AAAS

advances.sciencemag.org/cgi/content/full/6/48/eabc9863/DC1

Supplementary Materials for

Robust inference of positive selection on regulatory sequences in the human brain

Jialin Liu* and Marc Robinson-Rechavi*

*Corresponding author. Email: jialin.liu@unil.ch (J.L.); marc.robinson-rechavi@unil.ch (M.R.-R.)

Published 27 November 2020, *Sci. Adv.* **6**, eabc9863 (2020)
DOI: 10.1126/sciadv.abc9863

This PDF file includes:

Figs. S1 to S20
Tables S1 and S2

1 Supplementary figures

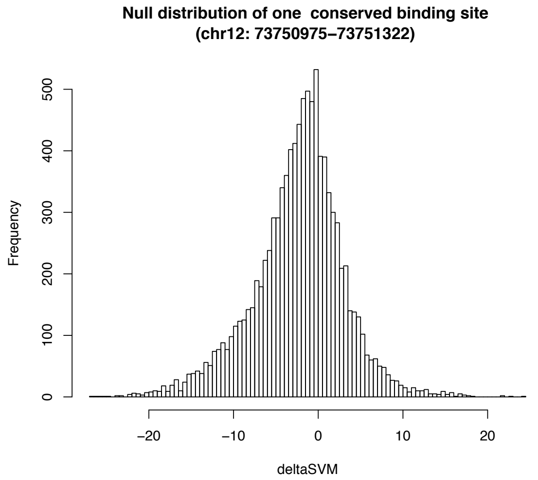

Figure S1: The null distribution of deltaSVM for a conserved biding site of mouse CEBPA

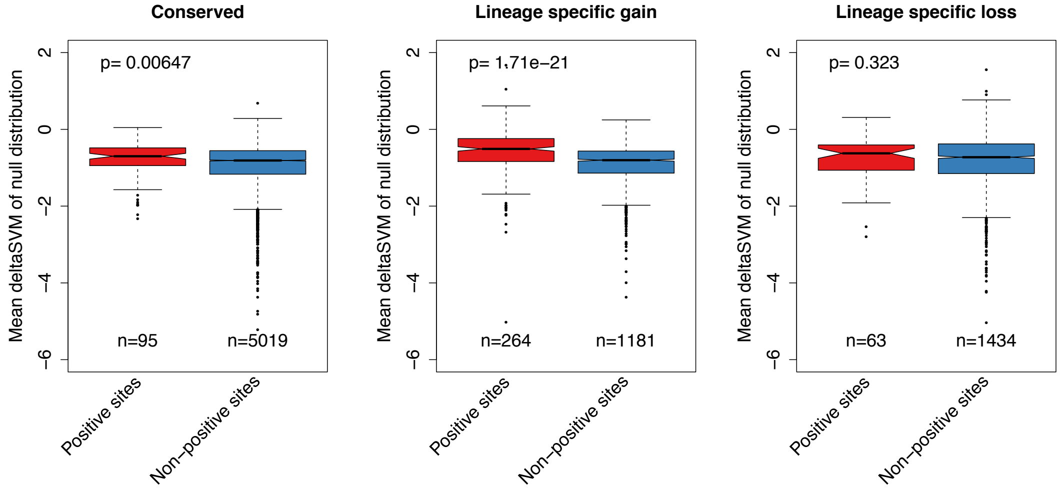

Figure S2: Comparison of mean deltaSVM of null distribution between positive sites and non-positive sites for mouse CEBPA

For each binding site, we first calculated its mean deltaSVM of null distribution. Then, we compared the mean values between positive sites and non-positive sites. The number of binding sites in each category is indicated below each box. The p-values from a Wilcoxon test comparing categories are reported above boxes. The lower and upper intervals indicated by the dashed lines ("whiskers") represent 1.5 times the interquartile range, or the maximum (respectively minimum) if no points are beyond 1.5 IQR (default behavior of the R function

boxplot). Positive sites are binding sites with evidence of positive selection (deltaSVM *p*-value < 0.01), non-positive sites are binding sites without evidence of positive selection.

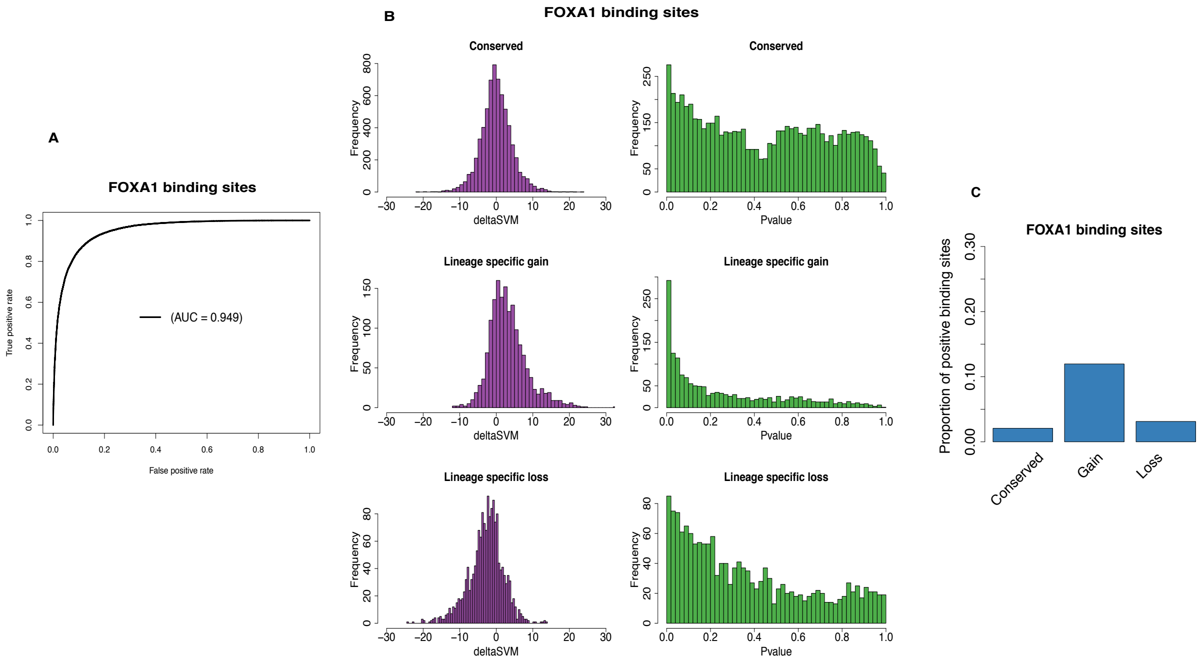

Figure S3: Mouse FOXA1 biding sites study

A. Receiver operating characteristic (ROC) curve for gkm-SVM classification performance on FOXA1 binding sites (5-fold cross validation). The AUC value represents the area under the ROC curve and provides an overall measure of predictive power.

B. The left hand graphs are the distributions of deltaSVM for conserved, gain, and loss binding sites. The right hand graphs are the distributions of deltaSVM p-values (test for positive selection) for conserved, gain, and loss binding sites.

C. The proportion of FOXA1 binding sites with evidence of positive selection in conserved, gain, and loss binding sites.

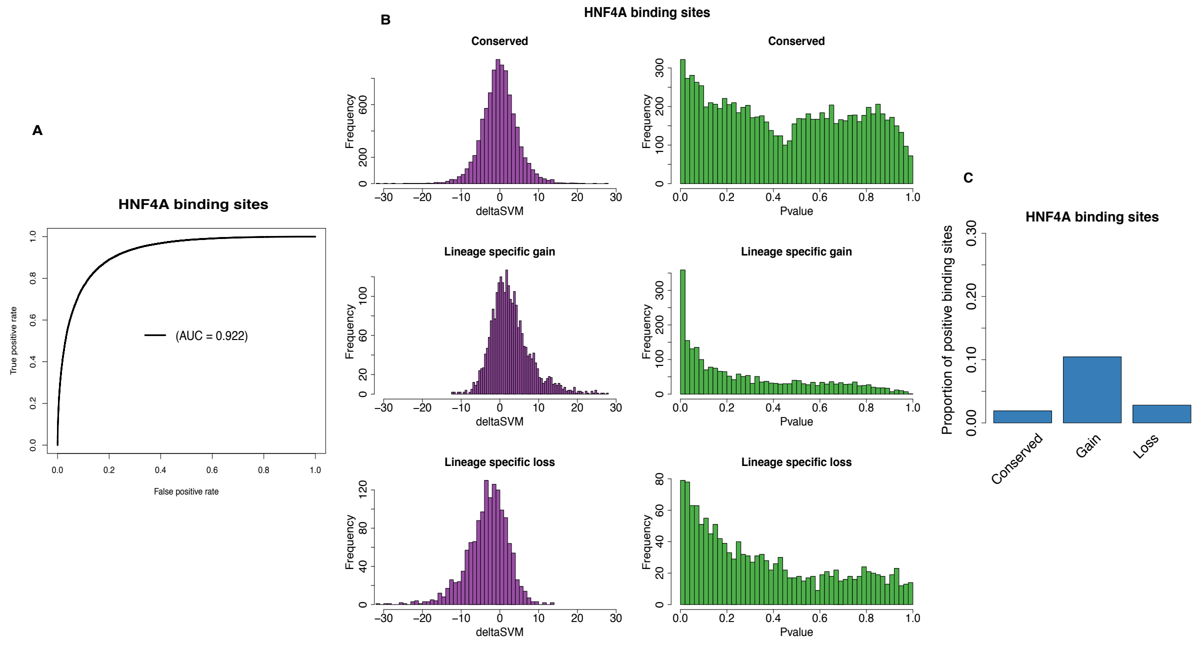

Figure S4: Mouse HNF4A biding sites study

A. Receiver operating characteristic (ROC) curve for gkm-SVM classification performance on HNF4A binding sites (5-fold cross validation). The AUC value represents the area under the ROC curve and provides an overall measure of predictive power.

B. The left hand graphs are the distributions of deltaSVM for conserved, gain, and loss binding sites. The right hand graphs are the distributions of deltaSVM p-values (test for positive selection) for conserved, gain, and loss binding sites.

C. The proportion of HNF4A binding sites with evidence of positive selection in conserved, gain, and loss binding sites.

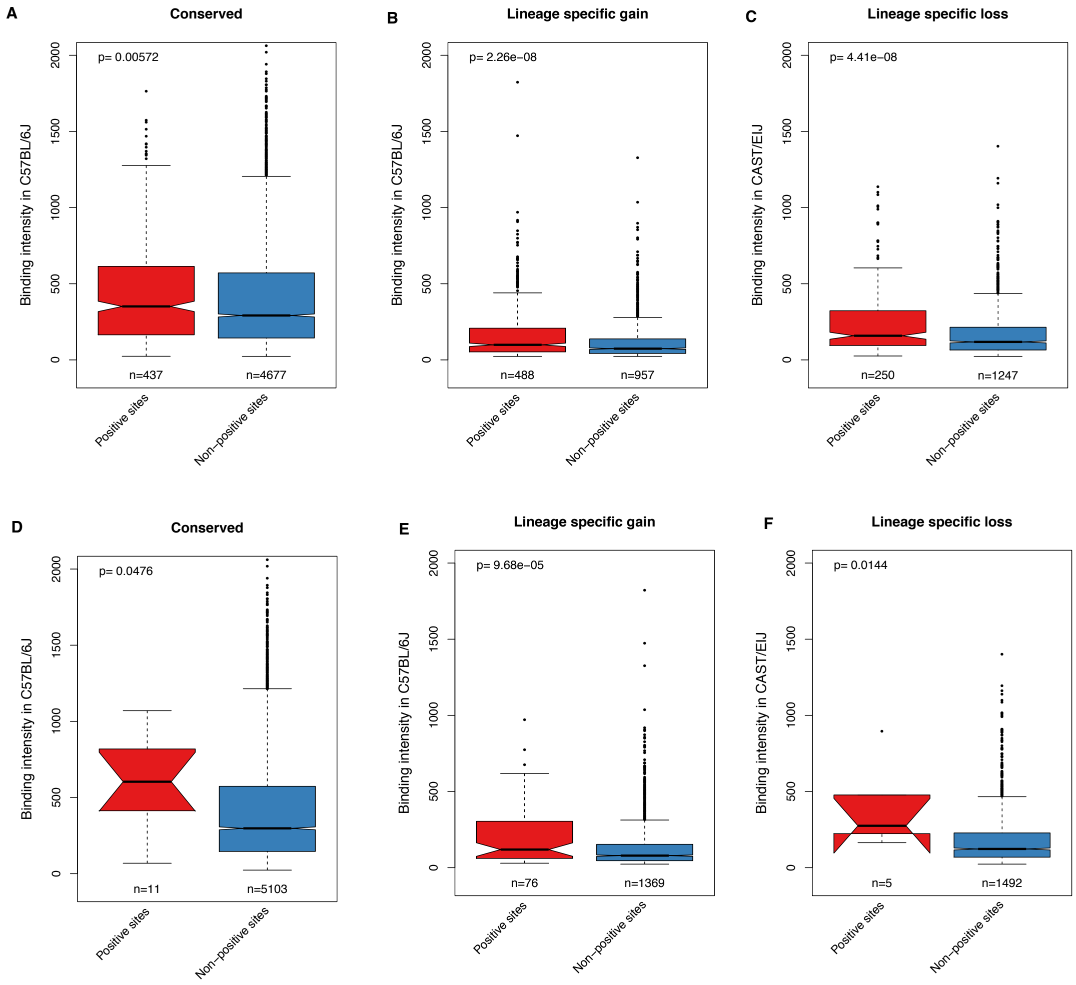

Figure S5: Comparison of biding intensity between positive sites and non-positive sites for mouse CEBPA

The number of binding sites in each category is indicated below each box. The p-values from a Wilcoxon test comparing categories are reported above boxes. The lower and upper intervals indicated by the dashed lines ("whiskers") represent 1.5 times the interquartile range, or the maximum (respectively minimum) if no points are beyond 1.5 IQR (default behavior of the R function boxplot).

A-C: Positive sites defined as deltaSVM with p-value < 0.05 instead of 0.01 in Figure 2E-G.

D-F: Positive sites defined as deltaSVM with p-value < 0.001 instead of 0.01 in Figure 2E-G.

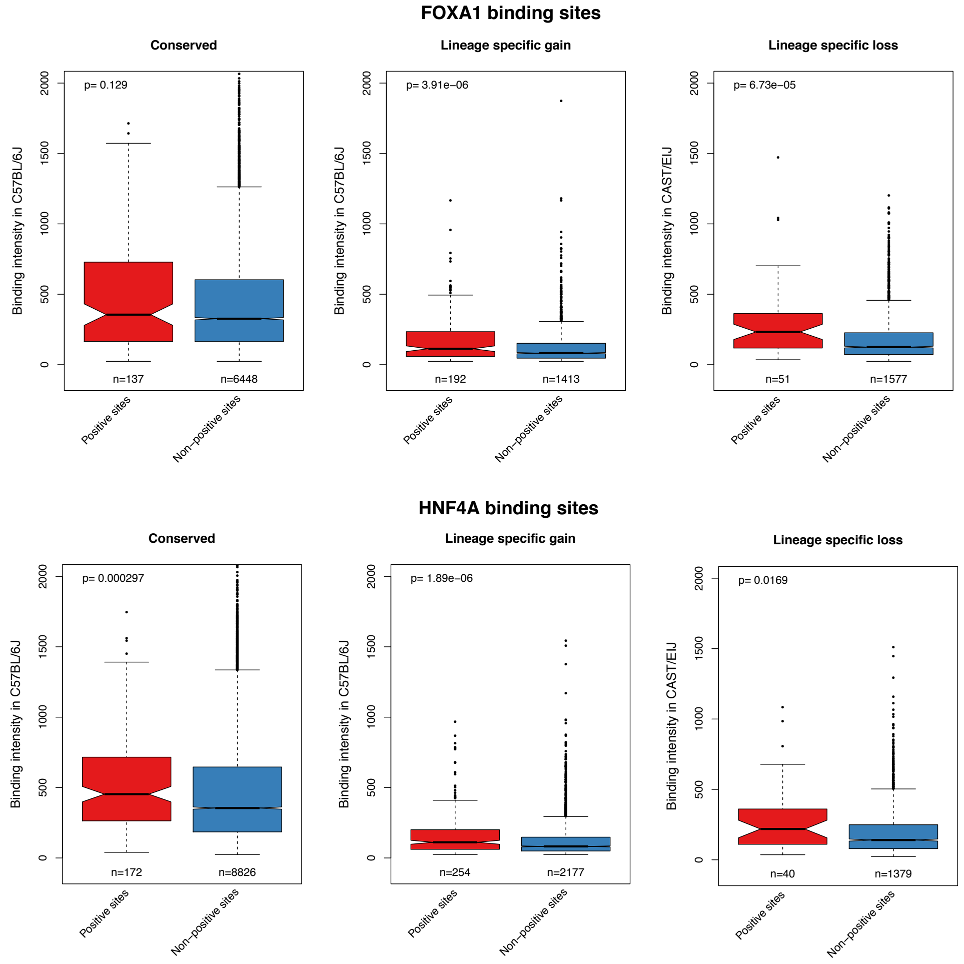

Figure S6: Comparison of biding intensity between positive sites and non-positive sites for mouse FOXA1 and HNF4A

The number of binding sites in each category is indicated below each box. The p-values from a Wilcoxon test comparing categories are reported above boxes. The lower and upper intervals indicated by the dashed lines ("whiskers") represent 1.5 times the interquartile range, or the maximum (respectively minimum) if no points are beyond 1.5 IQR (default behavior of the R function boxplot). Positive sites are binding sites with evidence of positive selection (deltaSVM p-value < 0.01), non-positive sites are binding sites without evidence of positive selection.

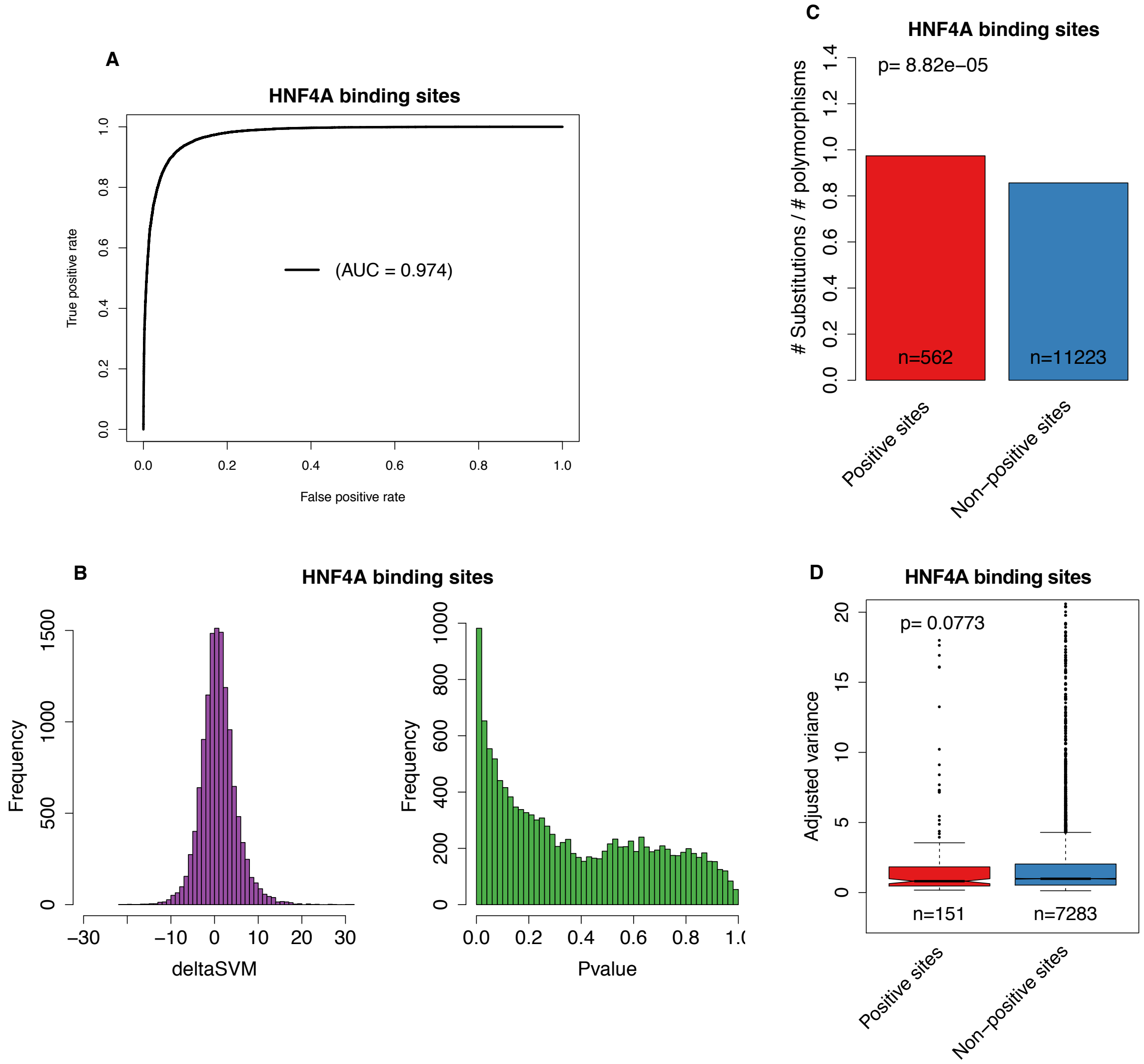

Figure S7: Human HNF4A biding sites study

A. Receiver operating characteristic (ROC) curve for gkm-SVM classification performance on HNF4A binding sites (5-fold cross validation). The AUC value represents the area under the ROC curve and provides an overall measure of predictive power.

B. The left graph is the distribution of deltaSVM. The right graph is the distribution of deltaSVM p-values (test for positive selection).

C. The ratio between the number of substitutions and the number of polymorphisms (SNPs) for HNF4A binding sites. Positive sites are binding sites with evidence of positive selection (deltaSVM p-value < 0.01), non-positive sites are binding sites without evidence of positive selection. The p-value from Fisher's exact test is reported above the bars.

D. Comparison of expression variance (adjusted variance) of putative target genes (closest gene to a TFBS) between positive sites and non-positive sites. The number of binding sites in each category is indicated below each box. The p-values from a Wilcoxon test comparing categories are reported above boxes. The lower and upper intervals indicated by the dashed lines ("whiskers") represent 1.5 times the interquartile range, or the maximum (respectively minimum) if no points are beyond 1.5 IQR (default behavior of the R function boxplot). Positive sites are binding sites with evidence of positive selection (deltaSVM p-value < 0.01), non-positive sites are binding sites without evidence of positive selection.

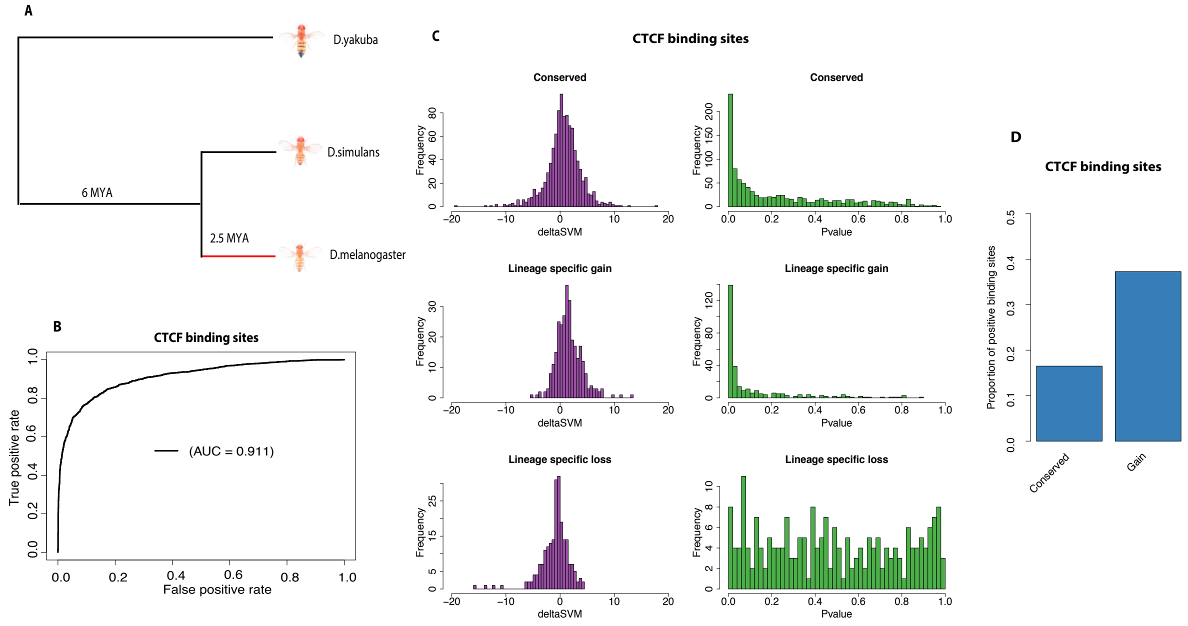

Figure S8: *D. melanogaster* CTCF biding sites study

A. Topological illustration of the phylogenetic relationships between the three *Drosophila* species used to detect positive selection on CTCF binding sites. We want to detect positive selection which occurred on the lineage of *D. melanogaster* after divergence from *D. simulans*, as indicated by the red branch. *D. yakuba* is the outgroup used to infer binding site sequence in the ancestor of *D. melanogaster* and *D. simulans*.

B. Receiver operating characteristic (ROC) curve for gkm-SVM classification performance on CEBPA binding sites (5-fold cross validation). The AUC value represents the area under the ROC curve and provides an overall measure of predictive power.

C. The left hand graphs are the distributions of deltaSVM for conserved, gain, and loss binding sites. The right hand graphs are the distributions of deltaSVM *p*-values (test for positive selection) for conserved, gain, and loss binding sites.

D. The proportion of CEBPA binding sites with evidence of positive selection in conserved and gain binding sites.

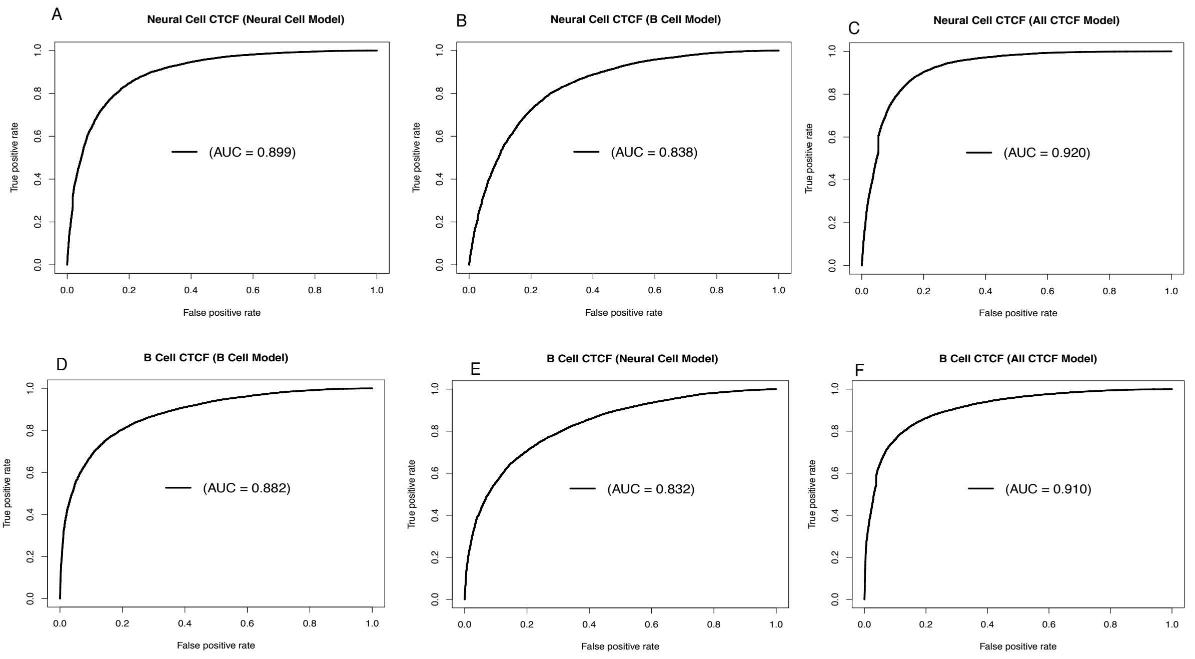

Figure S9: Receiver operating characteristic (ROC) curves for gkm-SVM classification performance on human CTCF binding sites

AUC values represent areas under the ROC curve and provide an overall measure of predictive power.

A. The results of a 5-fold cross validation on neural CTCF binding sites and matched random sequences.

B. The gkm-SVM trained in B cell used to predict neural CTCF binding sites and matched random sequences.

C. The gkm-SVM trained in all 29 tissues/cell types used to predict neural CTCF binding sites and matched random sequences.

D. The results of a 5-fold cross validation on B cell CTCF binding sites and matched random sequences.

E. The gkm-SVM trained in neural cell used to predict B cell CTCF binding sites and matched random sequences.

F. The gkm-SVM trained in all 29 tissues/cell types used to predict B cell CTCF binding sites and matched random sequences.

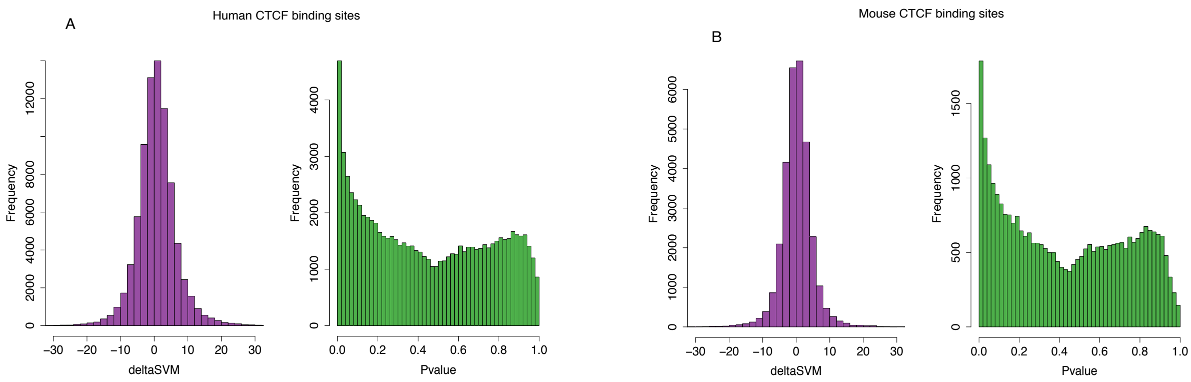

Figure S10: The distribution of deltaSVM and deltaSVM *p*-values for CTCF biding sites in human and mouse

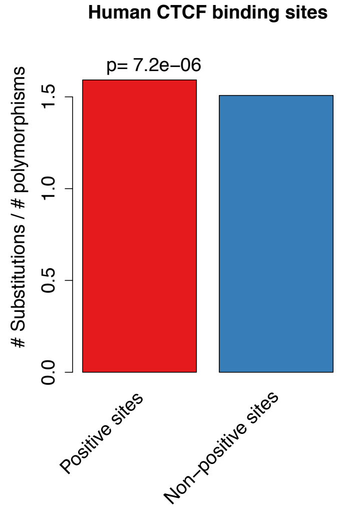

Figure S11: Ratio between the number of substitutions and the number of polymorphisms (SNPs) for human CTCF binding sites

Positive sites are binding sites with evidence of positive selection (deltaSVM *p*-value < 0.01), non-positive sites are binding sites without evidence of positive selection. The *p*-value from Fisher's exact test is reported above the bars.

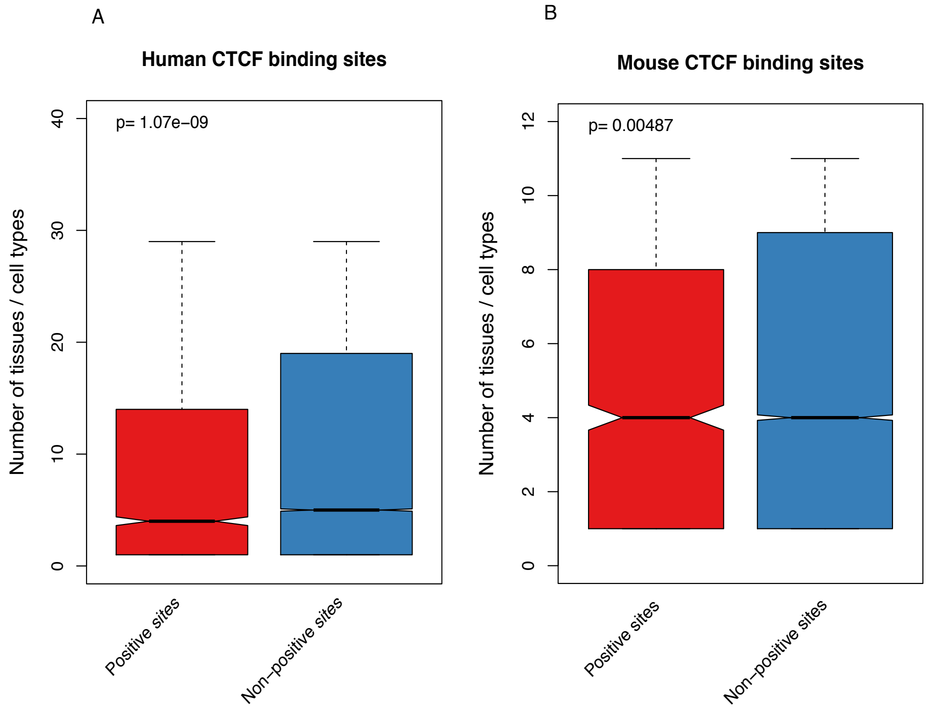

Figure S12: Comparison of the number of active tissues/cell types for CTCF biding sites between positive sites and non-positive sites

The *p*-values from a Wilcoxon test comparing categories are reported above boxes. The lower and upper intervals indicated by the dashed lines ("whiskers") represent 1.5 times the interquartile range, or the maximum (respectively minimum) if no points are beyond 1.5 IQR (default behavior of the R function boxplot). Positive sites are binding sites with evidence of positive selection (deltaSVM *p*-value < 0.01), non-positive sites are binding sites without evidence of positive selection.

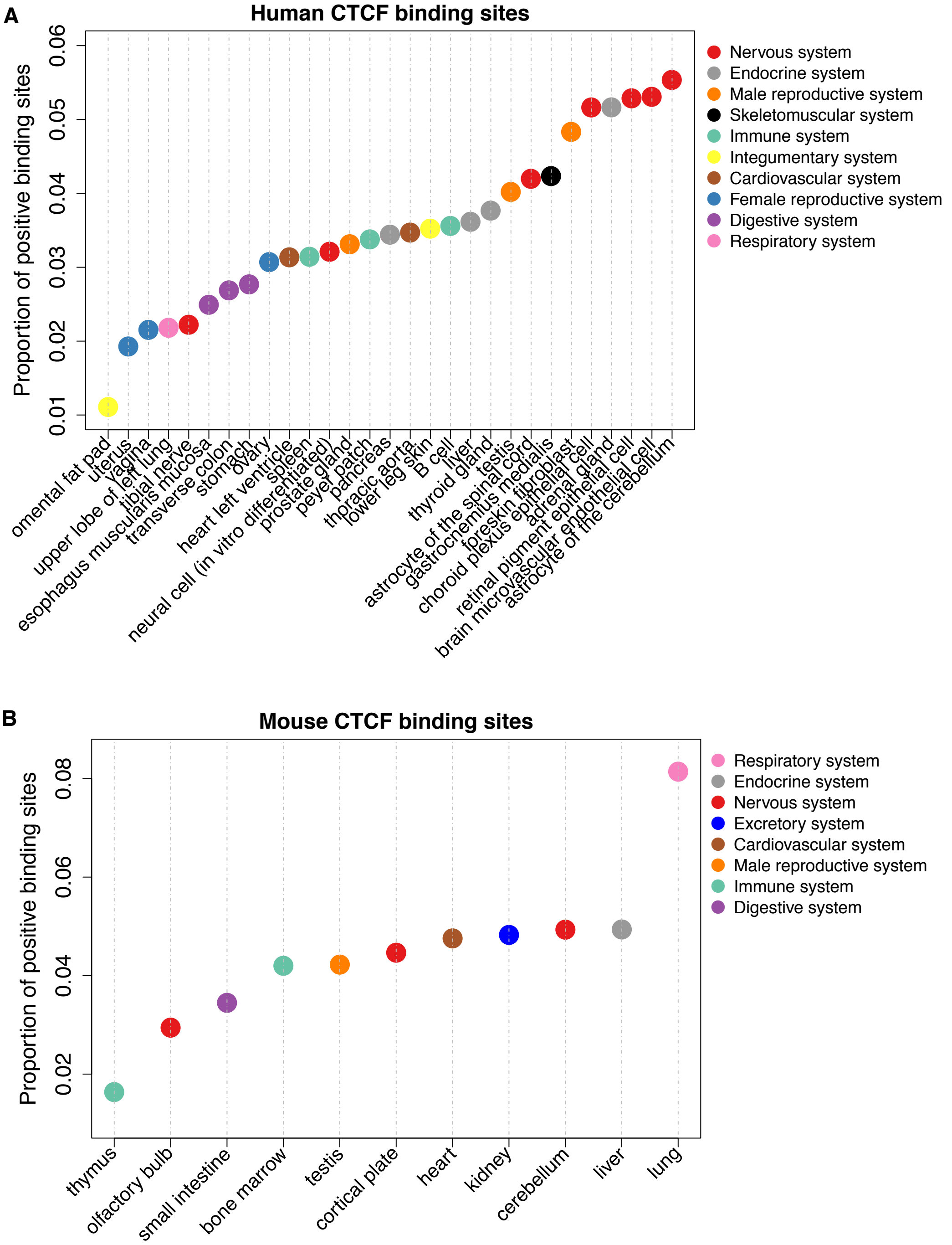

Figure S13: Proportion of positive CTCF binding sites in different tissues or cell types

Here, we only consider cell type or tissue specific CTCF binding sites. Positive binding sites are binding sites with evidence of positive selection (deltaSVM p-value < 0.01). Colors correspond to broad anatomical systems.

A. CTCF binding sites in 29 human tissues or cell types.

B. CTCF binding sites in 11 mouse tissues.

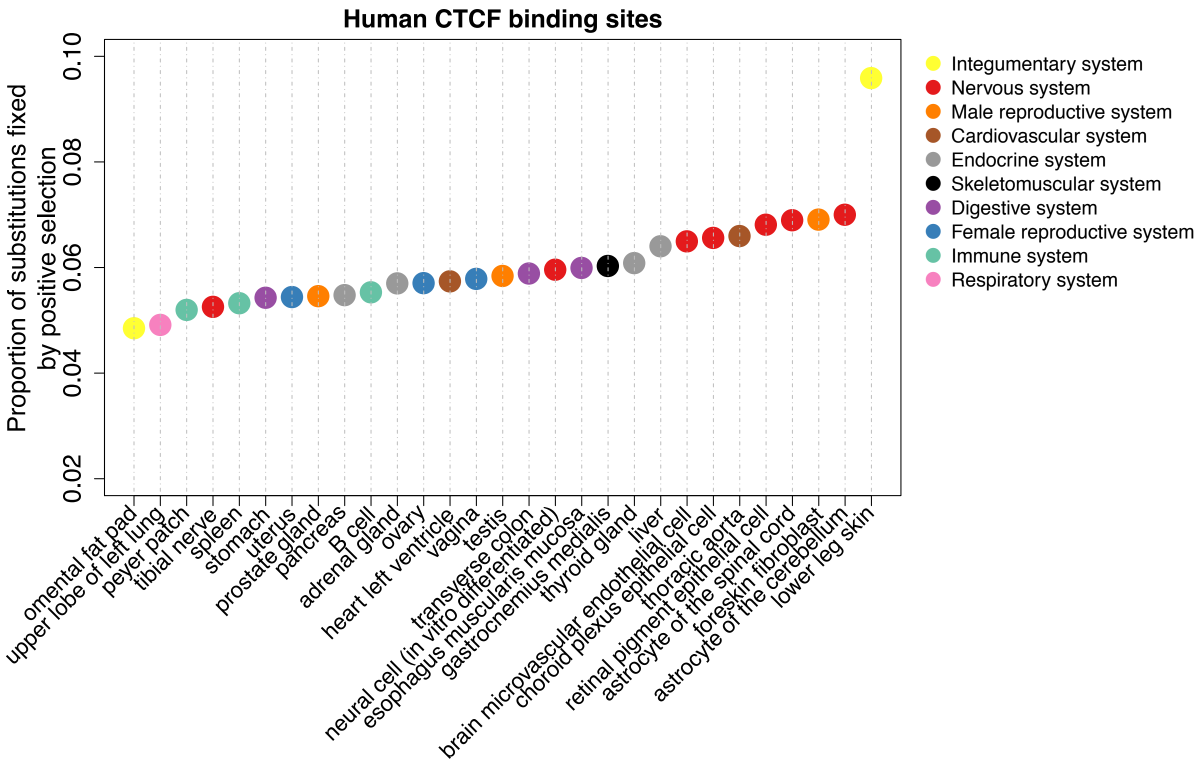

Figure S14: Proportion of substitutions fixed by positive selection in different tissues or cell types

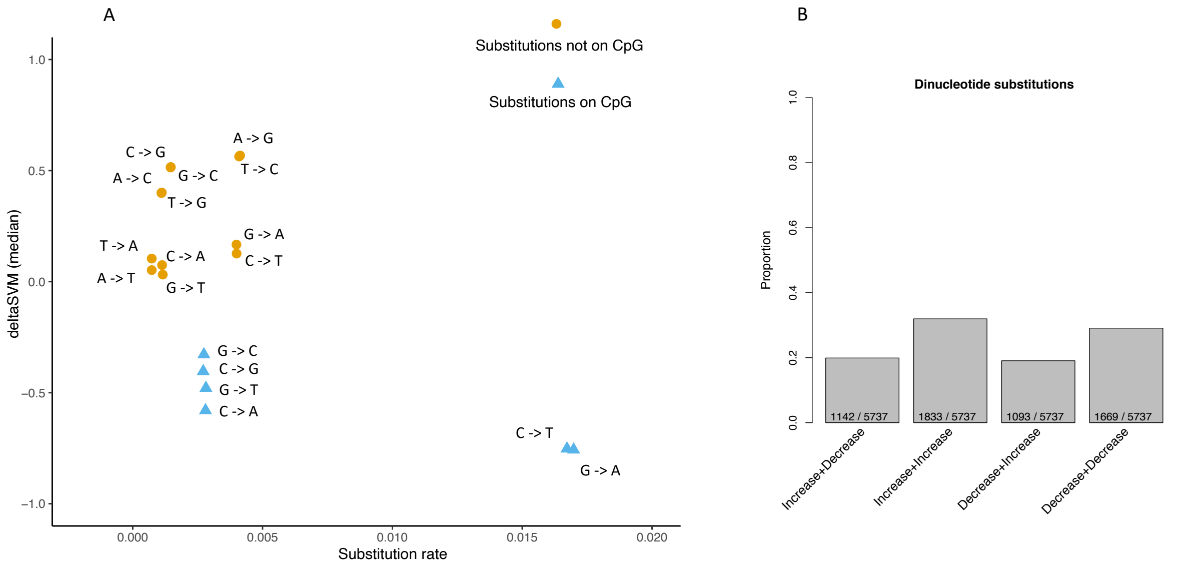

Figure S15: Substitution type, substitution rate and deltaSVM relationship

A. The x-axis is the substitution rate of different types of substitutions, the y-axis is the median deltaSVM of different types of substitutions.

B. The label below the bottom of each bar indicates the direction of binding affinity change

of two neighboring substitutions. For example, Increase+Decrease indicates the first substitution increases the binding affinity, but the second substitution decreases the binding affinity. the number of dinucleotide substitutions in each category and the total number of dinucleotide substitutions are indicated in the bottom of each bar.

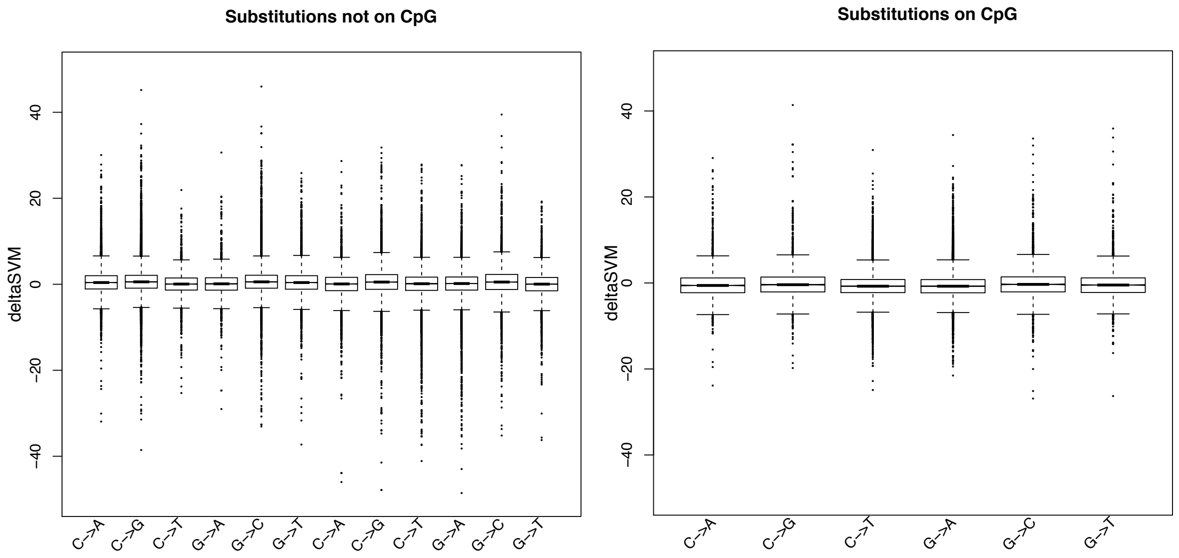

Figure S16: deltaSVM comparisons between different type of Substitutions

The lower and upper intervals indicated by the dashed lines ("whiskers") represent 1.5 times the interquartile range, or the maximum (respectively minimum) if no points are beyond 1.5 IQR (default behavior of the R function boxplot).

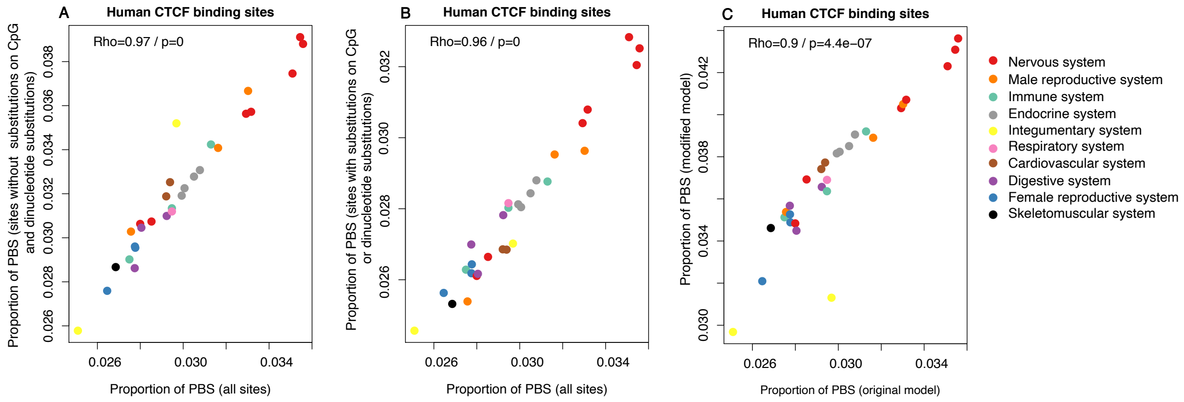

Figure S17: Correlation of PBS proportion between different analyses

PBS, the positive selection sites defined as (deltaSVM *p*-value <0.01).

A. The x axis is the proportion of PBS in different tissues and cell types for all sites, the y axis

is the proportion of PBS in different tissues and cell types for sites without substitutions on CpG and without dinucleotide substitutions.

B. x axis is the proportion of PBS in different tissues and cell types for all sites, y axis is the proportion of PBS in different tissues and cell types for sites with substitutions on CpG or with dinucleotide substitutions.

C. x axis is the proportion of PBS in different tissues and cell types from the original analysis, y axis is the proportion of PBS in different tissues and cell types for sites from the new analysis. In the new analysis, we excluded all CpG sequences and dinucleotide substitution sequences, and controlled the transition and transversion rate.

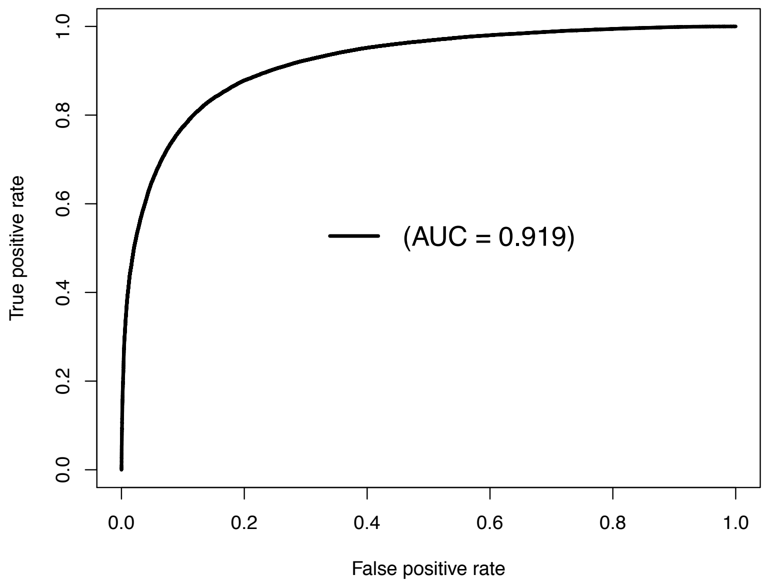

Figure S18: Receiver operating characteristic (ROC) curve for gkm-SVM classification performance on mouse CTCF binding sites

The result comes from a 5-fold cross validation on all CTCF binding sites from 11 tissues and matched random sequences. The AUC value represents areas under the ROC curve and provides an overall measure of predictive power.

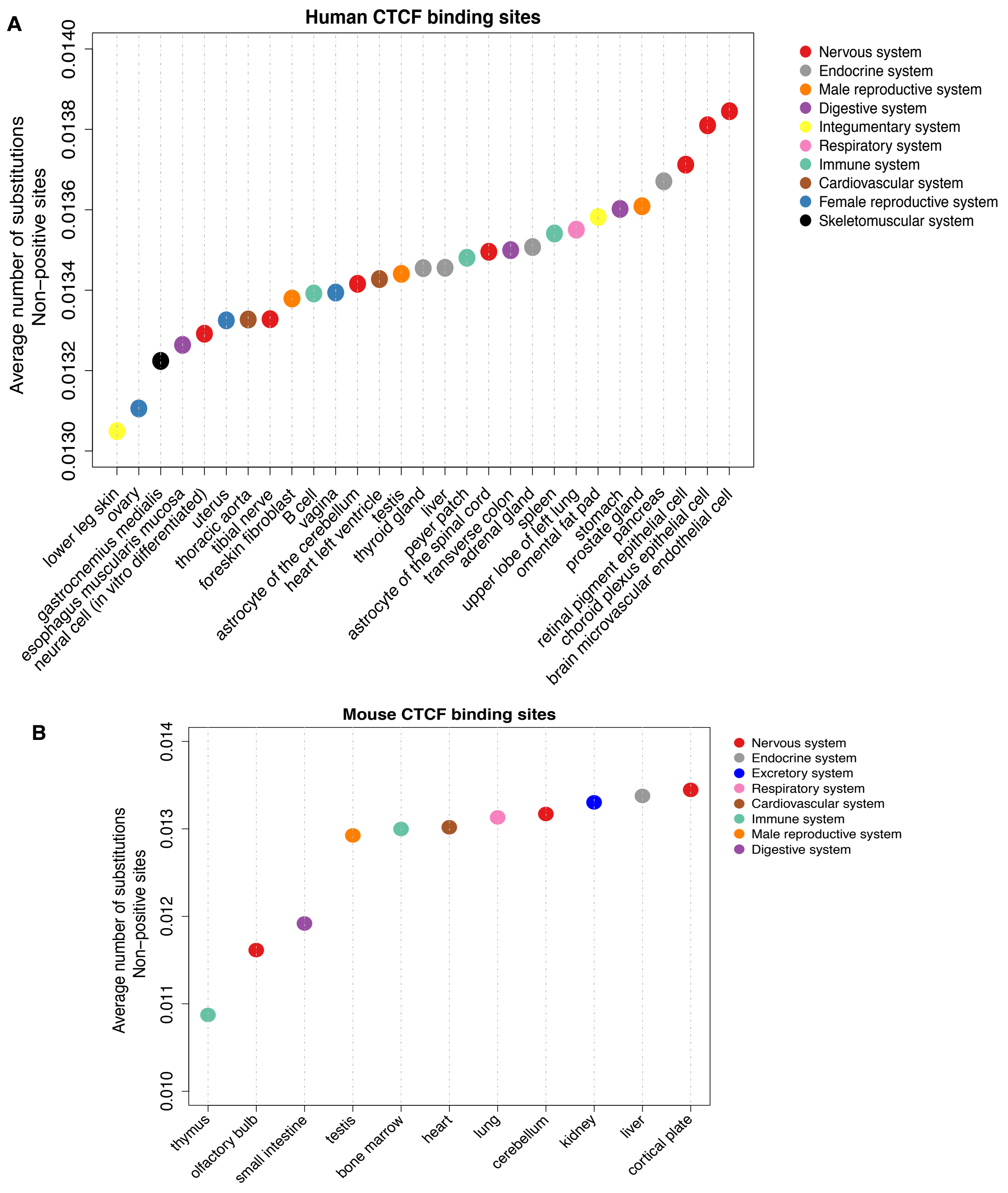

Figure S19: Average number of substitutions for non-positive CTCF binding sites in different tissues/cell types

Non-positive binding sites are binding sites without evidence of positive selection (deltaSVM p-value >= 0.01). For human, the number of substitutions measured by the pairwise whole genome alignments between human and chimpanzee. For mouse, the number of substitutions measured by the pairwise whole genome alignments between C57BL/6J and CAST/EiJ. For each binding site, the number of substitutions was normalized by the length of the binding site.

A. CTCF binding sites in 29 human tissues/cell types.

B. CTCF binding sites in 11 mouse tissues.

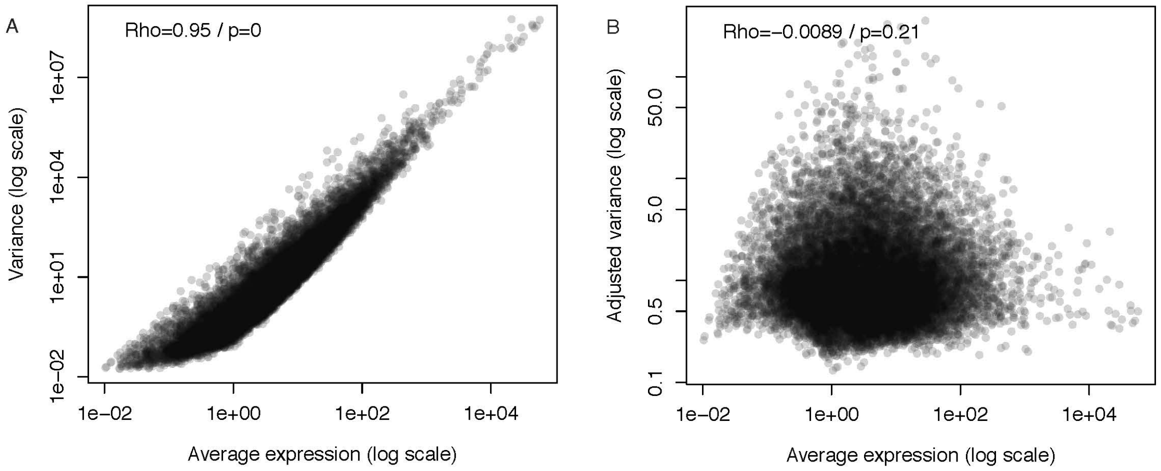

Figure S20: Relationship between mean expression and variance

A. Spearman's correlation between mean expression and variance.

B. Spearman's correlation between mean expression and adjusted variance.

2 Supplementary tables

Table S1: The number of peaks and average peak length for CEBPA, FOXA1 and HNF4A in three mouse species C57BL/6J, CAST/EiJ and SPRET/EiJ.

Factors / Species	CEBPA	FOXA1	HNF4A
**C57BL/6J**	41945 (270 bp)	50683 (276 bp)	68310 (298 bp)
**CAST/EiJ**	51057 (266 bp)	59780 (280 bp)	72257 (289 bp)
**SPRET/EiJ**	54035 (244 bp)	64027 (271 bp)	60142 (275 bp)

Table S2: The CTCF ChIP-seq datasets information for human and mouse.

Human			
tissue/cell type	Encode accession ID	# peaks	sample information
transverse colon	ENCFF547PLX	42837	male adult 37 years
stomach	ENCFF560VSK	24358	male adult 54 years
peyer patch	ENCFF777TZZ	44111	male adult 37 years
adrenal gland	ENCFF622LJD	38269	male adult 37 years
pancreas	ENCFF628TDS	44068	male adult 37 years
spleen	ENCFF459AHK	20634	male adult 37 years
upper lobe of left lung	ENCFF984EZB	44154	male adult 37 years
lower leg skin	ENCFF733VAF	6905	male adult 37 years
thyroid gland	ENCFF384ALJ	40010	male adult 54 years
gastrocnemius medialis	ENCFF101PDP	23000	male adult 54 years

heart left ventricle	ENCFF240UFV	44400	female adult 53 years
omental fat pad	ENCFF454PSG	7257	male adult 37 years
tibial nerve	ENCFF407FUL	38250	male adult 37 years
uterus	ENCFF282BOE	34171	female adult 53 years
ovary	ENCFF261BWI	25291	female adult 53 years
prostate gland	ENCFF899MQP	42180	male adult 37 years
testis	ENCFF432XLE	24328	male adult 37 years
liver	ENCFF690BYG	38845	male adult 32 years
B cell	ENCFF449NOT	52144	female adult 43 years
thoracic aorta	ENCFF330BPK	36852	male adult 37 years
astrocyte of the cerebellum	ENCFF660HHS	45500	primary cell
brain microvascular endothelial cell	ENCFF065LHJ	61680	primary cell
choroid plexus epithelial cell	ENCFF700ILD	62000	primary cell
astrocyte of the spinal cord	ENCFF312HCK	47893	primary cell
neuron (in vitro differentiated cells)	ENCFF618DDO	51087	in vitro differentiated cells
Esophagus muscularis mucosa	ENCFF735EHK	31886	male adult 32 years
retinal pigment epithelial cell	ENCFF139DOR	55106	primary cell
vagina	ENCFF176MPT	26446	female adult 53 years
foreskin fibroblast	ENCFF337WIE	48000	primary cell
Mouse			
tissue	Encode accession ID	# peaks	sample information
liver	ENCFF542WEE	37167	male adult (8 weeks)
lung	ENCFF605YVN	29061	male adult (8 weeks)
heart	ENCFF616HYA	35386	male adult (8 weeks)
kidney	ENCFF311HPG	37370	male adult (8 weeks)
bone marrow	ENCFF806PDR	27977	male adult (8 weeks)
cerebellum	ENCFF357KNB	32463	male adult (8 weeks)
cortical plate	ENCFF034VZI	32919	male adult (8 weeks)

olfactory bulb	ENCFF143RHK	14427	male adult (8 weeks)
small intestine	ENCFF319LOC	28414	male adult (8 weeks)
testis	ENCFF443TPY	24188	male adult (8 weeks)
thymus	ENCFF714WDP	20199	male adult (8 weeks)
